# Hepatic vein pressure predicts GFR in cirrhotic patients with hemodynamic kidney dysfunction

**DOI:** 10.14814/phy2.13301

**Published:** 2017-06-14

**Authors:** Neel Desai, Joel Neugarten, Mary Dominguez, Ladan Golestaneh

**Affiliations:** ^1^ Montefiore Medical Center/Albert Einstein College of MedicineDepartment of Medicine/Renal DivisionBronxNew York

**Keywords:** Cirrhosis, eGFR, hepatic vein pressure

## Abstract

The role of “nephrocongestion” in hemodynamic renal disease is understudied. Intra‐abdominal hypertension accompanies liver disease and renal disease. Our hypothesis states that in those patients with liver disease, hepatic vein pressure measured during a transjugular intrahepatic portosystemic shunt (TIPS) procedure reflects intra‐abdominal pressure and predicts estimated glomerular filtration rate (eGFR). We gathered data from our clinical database and chart review on a cohort of cirrhotic patients who received TIPS at Montefiore as part of their clinical care between 2004 and 2014. We evaluated association of demographic and measured variables with eGFR in those subjects without end‐stage renal disease (ESRD). Using multivariate regression, we examined the relationship between eGFR and hepatic vein pressure while adjusting for age, proteinuria, and ultrasound evidence for parenchymal kidney disease. The mean age of the subjects was 57 years old. Two thirds of the patients were male, 23% were White, and 20% were Black. A higher percentage of patients with chronic kidney disease (CKD), as determined by lower than 60 mL/min/1.73 m^2^, had proteinuria and ultrasound evidence for parenchymal kidney disease. A multivariate linear regression showed a significant and negative association between hepatic vein pressure and eGFR when adjusting for age, race, and proteinuria. Hepatic vein pressure is negatively and significantly associated with eGFR in those patients with liver failure. This finding has major implications for the way we evaluate hemodynamic renal disease.

## Introduction

Hemodynamic renal disease, also known as prerenal azotemia, is characterized by a reduction in glomerular filtration rate (eGFR) that is reversible and presumably a result of decreased renal perfusion (Schrier 2007; Schrier et al. 2012). Etiologies for hemodynamic renal disease include conditions wherein total body salt water is increased but “effective” intravascular volume is reduced. Examples include cardiorenal syndrome in heart failure (HF) and hepatorenal syndrome in cirrhosis (Schrier 2007; Schrier et al. 2012). The mechanism for renal dysfunction in these settings is yet to be fully elucidated. Recently, theories supporting a role for renal venous hypertension as a causal factor have emerged. A thorough understanding of hemodynamic mechanisms underlying renal dysfunction in these disorders is essential for proper diagnosis and has significant implications for management strategies.

There are multiple purported etiologies for kidney failure in the setting of liver cirrhosis (Epstein 1992, 1994; Schrier et al. 2012; Adebayo et al. 2015). The mechanisms by which intra‐abdominal hypertension (IAH) from ascites and portal hypertension cause hemodynamic renal disease are not well established, but possibilities include renal interstitial hypertension, efferent arteriolar hypertension, and afferent arteriolar vasoconstriction (Kostreva et al. 1980; Cade et al. 1987; Bloomfield et al. 1997; Chang et al. 2013; Adebayo et al. 2015). Studies have shown that a reduction in IAP below 17 with paracentesis is associated with an improvement in renal blood flow (RBF) and urine output in this setting (Cade et al. 1987; Chang et al. 2013; Adebayo et al. 2015). Further studies have shown that the insertion of a peritoneovenous shunt leads to improved RBF and renal function (Cade et al. 1987; Adebayo et al. 2015). Chang et al. (2013), in a murine model of cirrhosis, showed that an increase in IAP above 10 was associated with renal dysfunction. Biopsies from affected mice showed constricted tubular lumens and hyperemia of the interstitium (Chang et al. 2013). Artificially induced elevations in renal venous pressure were associated with decreases in urine output, and GFR in a number of animal models (Winton 1931, 1964; Gottschalk and Mylle 1956). Finally, Cade et al. (1987) were able to measure IVC, hepatic vein, and renal vein pressures before, during, and after large volume paracentesis in human subjects. They showed that the three parameters were almost identical at all intervals measured, and that a pressure decrease from 30 to 12 was associated with improved renal blood flow and GFR (Cade et al. 1987). A plausible link between intra‐abdominal venous pressure, hepatic vein pressure, renal vein pressure, and hemodynamic renal disease would dramatically inform our understanding of cirrhosis and hemodynamic renal disease.

We hypothesized that an increase in free hepatic venous pressure (FHVP) correlates with GFR in those patients with cirrhosis suspected of having hemodynamic renal disease. To test our hypothesis, we examined a cohort of cirrhotic patients who received transjugular intrahepatic portosystemic shunt (TIPS) procedures at Montefiore Medical Center between 2004 and 2014.

## Methods

We used “Clinical Looking Glass (CLG)”, Montefiore's clinical and claims database, to search for all TIPS procedures performed between the years 2004 and 2015. We used procedure codes utilized by our interventional radiology department. We identified a total of 439 procedures and utilized chart review to collect the following demographic variables: age, sex, race, history of diabetes mellitus (DM), history of hepatitis C, the most recent echocardiogram derived ejection fraction (EF) before the procedure, and the most recent serum creatinine and CLG derived estimated glomerular filtration rate (eGFR) using the Modification of Diet in Renal Disease (MDRD) formula. We also tallied use of loop diuretics in the 3 days prior to the index procedure, use of angiotensin converting enzyme inhibitors (ACEIs)/angiotensin receptor blockers (ARBs) in the preceding 3 days, evidence of renal parenchymal disease on most recent renal sonogram report (i.e., small kidney size, scarring, echogenicity), presence of proteinuria (greater than trace, where available) in most recent urinalysis, and hepatic wedge pressure and FHVP measurements during the TIPS procedure. We utilized renal ultrasound evidence for parenchymal renal disease and proteinuria as a way to refine our definition of hemodynamic renal disease. TIPS procedures were done in the interventional radiology suite, under fluoroscopy, and with conscious sedation, as clinically indicated. Measurement of hepatic vein and hepatic wedge pressures are done routinely in this setting. Patients with a history of end‐stage renal disease (ESRD) were excluded.

STATA version 14.0 was used for all data analyses. Bivariate analyses were performed to examine the association between eGFR, FHVP, ejection fraction (EF) from echocardiogram done closest in date to the TIPS procedure, and demographic attributes such as age, gender, and race. Those values for eGFR and FHVP that were over 3 standard deviations (outliers) from the mean were excluded in the testing. We used Pearson's correlation, *T*‐test, and one‐way ANOVA for those normally distributed continuous variables such as age, EF, eGFR, and FHVP. Nonparametric testing included Spearman's rho and Mann–Whitney tests. Chi‐square testing was used for all categorical variables.

A linear regression model was built with eGFR as the outcome variable and FHVP as the variable of interest. We used backward stepwise regression with a two‐sided *P*‐value of 0.1 to eliminate variables that were not significant after including only those variables that were significant from bivariate testing. The variables in the final model were FHVP, age, and proteinuria. Postestimation testing was carried out using residual testing and RVP plots. Interactions were tested between the variable of interest and each of the other variables in the model, and no significant interactions were found.

## Results

A total of 439 TIPS procedures were identified. The mean age of the cohort was 57 years old and 67% of the cohort was male. Twenty‐three percent of the patients were White, 20% were Black, 6% identified as Hispanic, and another 8.7% identified as multiracial (Table 1). Thirty percent of the cohort had CKD stage 3 or greater. Seventeen percent of all patients had evidence of parenchymal renal disease on renal sonogram, while 33.2% of the patients had proteinuria. The mean eGFR of the entire cohort was 84 mL/min/m^2^. In those patients identified as having CKD stage 3 or above, the prevalence of parenchymal renal disease was 25% and that of proteinuria was 55%. The mean ejection fraction for all subjects was 64% and the mean hepatic vein pressure was 14.0 mmHg.

**Table 1:** Demographic data for the cohort of patients who had a TIPS procedure at Montefiore Medical Center during the study period

Variable in total cohort (*N* = 439)	
Age (years), mean (±SD) (*n* = 438)	57.0 (11.4)
Gender (%) (*n* = 427)	
Female	141 (33.0)
Male	286 (67.0)
Race (%) (*n* = 439)	
White	102 (23.2)
Black	88 (20.1)
Hispanic	29 (6.6)
Multiracial	38 (8.7)
Other	182 (41.5)
Calculated GFR (mL/min/1.73 m^2^), mean (SD) (*n* = 430)	84 (40.0)
Presence of CKD (%) (*n* = 439)	
No CKD	307 (70.0)
Stage 3	100 (22.8)
Stage 4	16 (3.6)
Stage 5	16 (3.6)
Evidence of parenchymal disease on renal sono (%) (*n* = 301)	52 (17.3)
Patients with proteinuria (%) (*n* = 310)	103 (33.2)
Mean baseline EF (± SD) (%) (*n* = 300)	64.3 (0.1)
FHVP (mmHg), mean (± SD) (*n* = 200)	14.2 (6.4)

We examined bivariate associations of variables with eGFR and found that with every year increase in age there is a negative 0.72 difference in eGFR, and for every 1 mmHg increase in hepatic vein pressure there is a negative 0.67 difference in eGFR, but the latter did not reach statistical significance (Table 2). There was no difference in eGFR between genders, White patients had lower eGFRs and patients with proteinuria and ultrasound evidence for kidney disease had significantly lower eGFRs (Table 1).

**Table 2:** Bivariate associations of study variables with eGFR

Variable	Association with eGFR		*P*
Age (*n* = 416) for every 1 year increase	−0.72		<0.001
Ejection fraction (*n* = 283) for every 1% increase	0.03		0.65
FHVP (*n* = 188) for every one mmHg increase	−0.67		0.06
Mean eGFR by gender (*N* = 400)	Female 78.1 (3.0)	Male 79.5 (2.0)	

**Figure 1:**
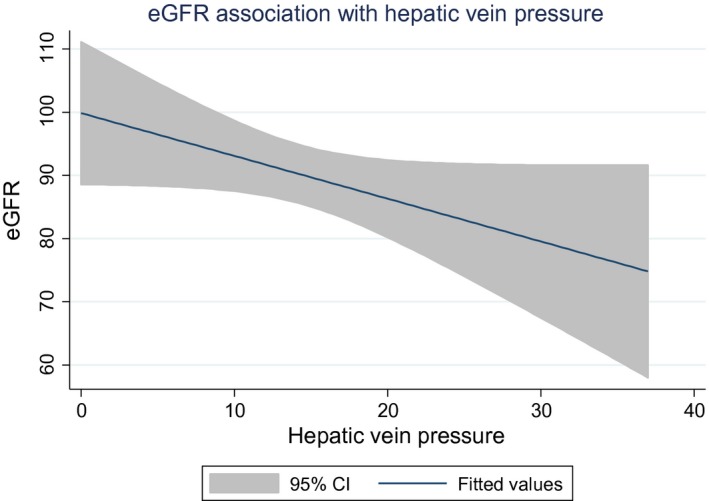
Fitted line representation of eGFR and free hepatic vein pressure (*n* = 176).

**Figure 2:**
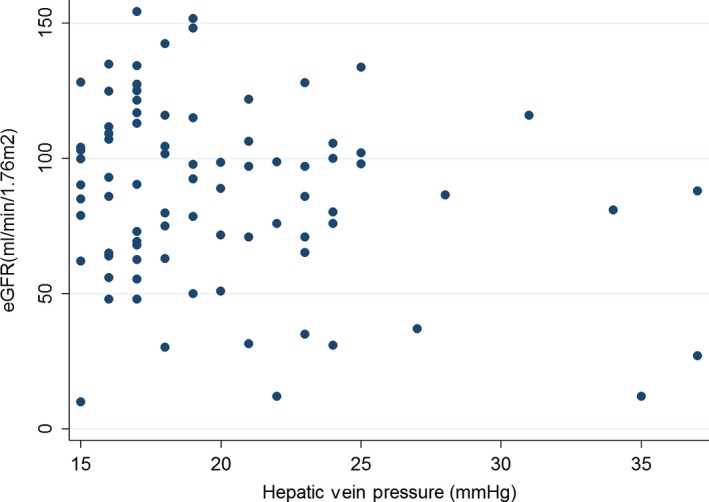
Scatter plot of association between eGFR and free hepatic vein pressure in those with FHVP >15.0 mmHg (*n* = 117).

A multivariate linear regression model included 176 data points. For every 1 mmHg increase in hepatic vein pressure there is a negative 0.75 mL/min/1.73 m^2^ difference in GFR at *P* = 0.02. The other two variables significantly associated with GFR in the final model were age and the presence of proteinuria. Every 1 year increase in age was associated with a negative 0.87 mL/min/1.73 m^2^ difference in eGFR which is significant at *P* < 0.001, and with a transition from no proteinuria to having trace proteinuria or greater is associated with a negative 18.3 mL/min/1.73 m^2^ difference in eGFR with *P* = 0.01 (Fig. 1). Furthermore, univariate analysis showed that the association between FHVP and eGFR is significant only above FHVP of 10 mmHg, below which there is a nonsignificant association. The association between eGFR and FHVP seems linear above FHVP of 15 mmHg (Fig. 2).

## Discussion

Our data suggest that in patients with cirrhosis undergoing TIPS there is a significant negative association between FHVP and eGFR, when adjusting for proteinuria and age. This association was significant only at the FHVP level of 10 mmHg or greater. IAH is defined above an IAP of 12 mmHg. These results suggest a role for venous congestion in the pathophysiology of hemodynamic renal disease, in the setting of severe liver failure. They also suggest that venous congestion affects renal hemodynamics beyond a certain threshold.

In the cardiorenal literature, venous congestion's role in decreasing eGFR has been suggested. Although mechanisms for renal hypoperfusion in the setting of liver failure have been proposed including reflex vasoconstriction in response to a hyperdynamic circulation and the effects of hormonal and vasoactive mediators, renal venous congestion and IAH's contributions also merits attention (Epstein 1992, 1994; Umgelter et al. 2009; Mohmand and Goldfarb 2011). Other cardiorenal studies showed that renal venous congestion resulted in neurohormonal manipulation of the renal arterial perfusion pressure via increased flow in the efferent arteriole, and this mechanism affected GFR (Adebayo et al. 2015). Our study shows that hepatic venous pressure exceeding 10 mmHg is the threshold after which higher hepatic vein pressure correlates negatively with eGFR. In studies by Ichikawa and Brenner, the researches manipulated aortic and venous pressures to examine intraglomerular hemodynamics (Blake 1951; Ichikawa and Brenner 1980; Bloomfield et al. 1997). Efferent arteriolar hypertension was associated with glomerulotubular balance and was thought to be a result of peritubular capillary starling forces and their effects on the proximal tubular reabsorptive capacity (Blake 1951; Bloomfield et al. 1997). The relationship between efferent arteriolar pressure and glomerular capillary pressures seems to be linear without a threshold effect. Early studies done on dog kidneys by Winton, however, showed that a minimum rise of 20 mmHg in venous pressure was required for a decrease in urine flow to occur, referring to threshold effect (Winton 1931). There is sensitivity to arterial flow variations at higher renal venous pressures as well, such that a smaller decrease in renal perfusion pressure results in a decrement in urine flow (Cade et al. 1987; Chang et al. 2013).

Our data speak to the prevalence of IAH in this cohort as evidence by a high mean FHVP, and to the detrimental role of renal venous hypertension in liver related kidney disease. This is important because it leads to reevaluation of our understanding of hemodynamic renal disease in the setting of heart failure, liver failure, and IAH. Portal hypertension occurs where there is resistance in portal blood flow, and occurs most frequently in the setting of liver cirrhosis. Changes in the liver architecture produce structural barriers to hepatic sinusoidal blood flow which in turn increase pressure in the portal system (Epstein 1994; Mohmand and Goldfarb 2011). Portal hypertension is accompanied by increased cardiac output to compensate for the concomitant vasodilatation (Epstein 1992, 1994; Mohmand and Goldfarb 2011). The hepatic venous pressure gradient quantifies the degree of portal hypertension. It is the difference between inferior vena cava pressure and the hepatic vein wedge pressure (meant to approximate portal pressure). Kostreva et al. induced increases in hepatic venous pressure by vena cava ligation in anesthetized dogs and showed a reflexive increase in renal sympathomimetic activity followed by renal vasoconstriction (Cheatham 2009). In animal studies with hepatic congestion induced by IVC constriction, renal vein pressures were shown to increase in parallel with IVC pressures (Winton 1931; Gottschalk and Mylle 1956; Kostreva et al. 1980; Priebe et al. 1980). Upregulation of sympathomimetic activity were observed, as was a reduction in eGFR (Kostreva et al. 1980; Priebe et al. 1980). Gottschalk and Mylle (1956) showed that elevation of renal venous pressure beyond a certain threshold of intratubular and peritubular capillary pressures created a decrease in urinary flow rate. In fact venous congestion resulted in an increase in kidney size, an increase in peritubular capillary pressure, and compression of the tubules. By way of ligature‐induced renal vein constriction, they also demonstrated a correlation between high renal vein pressures and intratubular and peritubular capillary pressures (Eggleton et al. 1940; Winton 1951; Gottschalk and Mylle 1956). At high levels of venous pressure, intratubular fluid movement decreased significantly suggesting a decrease in glomerular filtration (Eggleton et al. 1940; Winton 1951). This phenomenon was later showed to be particularly true in saline loaded animals with intravascular congestion. Furthermore, experiments done in dogs showed that elevation of renal venous pressure to greater than 20–55 cm of water caused diminution in urine volume and a reduction in GFR (Eggleton et al. 1940; Blake 1951). One must be cautious with using animal studies and single nephron models as a reference point for patients with complex systemic physiologic processes. These models only form a foundation to begin to understand human renal physiology.

A reduction in IAP below 17 via paracentesis led to improved renal function in cirrhotic patients (Adebayo et al. 2015). IAH correlates with renal dysfunction and IAH has increasingly been recognized as a factor correlating with significant morbidity and mortality in the critically ill hospitalized patients over the past decade (Doty et al. 2000; Malbrain et al. 2006). Animal data and human observational studies dating back over 100 years indicate that oliguria and acute kidney injury (AKI) are frequent complications of IAH/ACS (Kostreva et al. 1980; Richards et al. 1983; Cheatham 2009). The mechanism by which renal impairment occurs is thought to be mediated via renal vein compression which impairs renal venous drainage and causes renal venous congestion (Bloomfield et al. 1997; Doty et al. 1999, 2000). In turn, renal venous congestion increases intra‐ and peritubular capillary pressure (Doty et al. 1999, 2000). By raising renal interstitial pressure and compressing ureters, elevated intra‐abdominal pressure may also increase intratubular and ultimately glomerular capillary hydrostatic pressure which in turn would reduce the transglomerular filtration gradient and eGFR (Bloomfield et al. 1997; Doty et al. 1999, 2000; Malbrain et al. 2006; Mohmand and Goldfarb 2011). Increased renal interstitial pressure may also activate the renin–angiotensin axis, stimulate the sympathetic nervous system, promote release of inflammatory cytokines, and induce tubulointerstitial inflammation and hypoxia (Le Roith et al. 1982; Bloomfield et al. 1997; Mohmand and Goldfarb 2011). Systemic venous congestion has been shown to correlate with endothelial stretch which in turn may contribute to endothelial dysfunction, reduced nitric oxide bioavailability, activation of neurohormonal pathways, and increased production of inflammatory cytokine and reactive oxygen species (Le Roith et al. 1982; Bloomfield et al. 1997; Doty et al. 2000). Mullens et al. (2008b) studied patients admitted to a specialized ICU for management of heart failure and found a high prevalence of elevated IAP and documented a correlation between elevated IAP and renal dysfunction. Reduction in IAP correlated with improved renal function in those with baseline elevated values. In contrast, there was no correlation between improvement in renal function on the one hand and changes in indices of right‐ or left‐sided cardiac function or filling pressures (mean arterial pressure, cardiac index, pulmonary capillary wedge pressure, or central venous pressure). In another group of patients presenting with ADHF and ascites, and studied by the same investigators, abdominal decompression by paracentesis led to improved renal function in the absence of changes in systemic blood pressure, PCWP, or CI (Mullens et al. 2008a). Finally, Umgelter et al. (2009) showed that after paracentesis there was a significant decrease in IAP and systemic vascular resistance index and a significant increase in creatinine clearance and urine output. These data are important to consider because they make the argument for IAP's role in renal venous congestion and dysfunction more plausible.

In our studies, the mean EF was higher than normal in this cirrhotic cohort, which is consistent with the vasodilated state described in liver failure; however, the EF did not meaningfully change the association between eGFR and FHVP in our model. Importantly, GFR did not associate with EF in the multivariate regression model, but did associate with age, evidence for parenchymal renal disease, and FHVP. In the overall cohort, age, proteinuria, and ultrasound evidence for renal disease associated significantly with eGFR. This was reassuring since these variables are established risk factors for renal disease, which validates our regression model. The prevalence of proteinuria and abnormal renal sonogram findings was higher in the CKD cohort further validating our data.

There were many limitations to our study. The sample size was small, and the study design was cross‐sectional and did not elucidate dynamic changes in eGFR with increasing central vein pressures, thus limiting causal inference. The measured eGFRs did not necessarily correspond to the day of the procedure, but were the last available measurements before the TIPS. MDRD was used to calculate eGFR which is not very accurate for eGFR greater than 60 mL/min/m^2^. Furthermore, our database, though comprehensive, may have hidden important confounders such as systemic hemodynamics, nephrotoxic medications, and other potential determinants of renal function.

## Conclusion

Our study is the first to show a significant association between hepatic vein pressures and eGFR in a cohort of cirrhotic patients undergoing TIPS. This association supports a role for venous congestion in the pathophysiology of hemodynamic renal disease in cirrhosis in humans. Our findings support a novel and important approach to evaluating the pathophysiology of hemodynamic renal disease in the setting of intravascular volume overload, and call into question the classic description of renal vasoconstriction as the sole mechanistic explanation. We advocate for a novel, more inclusive, approach to the complex interplay of hemodynamic renal disease and one that holds promise for new therapeutic options.

## Conflict of Interest

None declared.

## References

[phy213301-bib-0001] Adebayo, D. , V. Morabito , A. Davenport , and R. Jalan . 2015 Renal dysfunction in cirrhosis is not just a vasomotor nephropathy. Kidney Int. 87:509–515. 25296092 10.1038/ki.2014.338 PMC4346614

[phy213301-bib-0002] Blake, W. D. 1951 Effect of exercise and emotional stress on renal hemodynamics, water and sodium excretion in the dog. Am. J. Physiol. 165:149–157. 14829584 10.1152/ajplegacy.1951.165.1.149

[phy213301-bib-0003] Bloomfield, G. L. , C. R. Blocher , I. F. Fakhry , D. A. Sica , and H. J. Sugerman . 1997 Elevated intra‐abdominal pressure increases plasma renin activity and aldosterone levels. J. Trauma 42:997–1004; discussion 1004‐1005. 9210531 10.1097/00005373-199706000-00002

[phy213301-bib-0004] Cade, R. , H. Wagemaker , S. Vogel , D. Mars , D. Hood‐Lewis , M. Privette , et al. 1987 Hepatorenal syndrome. Studies of the effect of vascular volume and intraperitoneal pressure on renal and hepatic function. Am. J. Med. 82:427–438. 3548346 10.1016/0002-9343(87)90442-6

[phy213301-bib-0005] Chang, Y. , X. Qi , Z. Li , F. Wang , S. Wang , Z. Zhang , et al. 2013 Hepatorenal syndrome: insights into the mechanisms of intra‐abdominal hypertension. Int. J. Clin. Exp. Pathol. 6:2523–2528. 24228115 PMC3816822

[phy213301-bib-0006] Cheatham, M. L. 2009 Abdominal compartment syndrome: pathophysiology and definitions. Scand. J. Trauma Resusc. Emerg. Med. 17:10. 19254364 10.1186/1757-7241-17-10 PMC2654860

[phy213301-bib-0007] Doty, J. M. , B. H. Saggi , H. J. Sugerman , C. R. Blocher , R. Pin , I. Fakhry , et al. 1999 Effect of increased renal venous pressure on renal function. J. Trauma 47:1000–1003. 10608524 10.1097/00005373-199912000-00002

[phy213301-bib-0008] Doty, J. M. , B. H. Saggi , C. R. Blocher , I. Fakhry , T. Gehr , D. Sica , et al. 2000 Effects of increased renal parenchymal pressure on renal function. J. Trauma 48:874–877. 10823530 10.1097/00005373-200005000-00010

[phy213301-bib-0009] Eggleton, M. G. , J. R. Pappenheimer , and F. R. Winton . 1940 The relation between ureter, venous, and arterial pressures in the isolated kidney of the dog. J. Physiol. 99:135–152. 16995228 10.1113/jphysiol.1940.sp003886 PMC1394060

[phy213301-bib-0010] Epstein, M. 1992 The hepatorenal syndrome–newer perspectives. New England J. Med. 327:1810–1811. 1435935 10.1056/NEJM199212173272509

[phy213301-bib-0011] Epstein, M. 1994 Hepatorenal syndrome: emerging perspectives of pathophysiology and therapy. J. Am. Soc. Nephrol. 4:1735–1753. 8068872 10.1681/ASN.V4101735

[phy213301-bib-0012] Gottschalk, C. W. , and M. Mylle . 1956 Micropuncture study of pressures in proximal tubules and peritubular capillaries of the rat kidney and their relation to ureteral and renal venous pressures. Am. J. Physiol. 185:430–439. 13327061 10.1152/ajplegacy.1956.185.2.430

[phy213301-bib-0100] Ichikawa, I. , and B. M. Brenner . 1980 Importance of efferent arteriolar vascular tone in regulation of proximal tubule fluid reabsorption and glomerulotubular balance in the rat. J. Clin. Invest. 65:1192–1201. 7364945 10.1172/JCI109774 PMC371453

[phy213301-bib-0013] Kostreva, D. R. , A. Castaner , and J. P. Kampine . 1980 Reflex effects of hepatic baroreceptors on renal and cardiac sympathetic nerve activity. Am. J. Physiol. 238:R390–R394. 7377377 10.1152/ajpregu.1980.238.5.R390

[phy213301-bib-0014] Le Roith, D. , H. Bark , M. Nyska , and S. M. Glick . 1982 The effect of abdominal pressure on plasma antidiuretic hormone levels in the dog. J. Surg. Res. 32:65–69. 6172664 10.1016/0022-4804(82)90186-x

[phy213301-bib-0015] Malbrain, M. L. , M. L. Cheatham , A. Kirkpatrick , M. Sugrue , M. Parr , J. De Waele , et al. 2006 Results from the international conference of experts on intra‐abdominal hypertension and abdominal compartment syndrome. I. Definitions. Intensive Care Med. 32:1722–1732. 16967294 10.1007/s00134-006-0349-5

[phy213301-bib-0016] Mohmand, H. , and S. Goldfarb . 2011 Renal dysfunction associated with intra‐abdominal hypertension and the abdominal compartment syndrome. J. Am. Soc. Nephrol. 22:615–621. 21310818 10.1681/ASN.2010121222

[phy213301-bib-0017] Mullens, W. , Z. Abrahams , G. S. Francis , D. O. Taylor , R. C. Starling , and W. H. Tang . 2008a Prompt reduction in intra‐abdominal pressure following large‐volume mechanical fluid removal improves renal insufficiency in refractory decompensated heart failure. J. Card Fail. 14:508–514. 18672199 10.1016/j.cardfail.2008.02.010

[phy213301-bib-0018] Mullens, W. , Z. Abrahams , H. N. Skouri , G. S. Francis , D. O. Taylor , R. C. Starling , et al. 2008b Elevated intra‐abdominal pressure in acute decompensated heart failure: a potential contributor to worsening renal function? J. Am. Coll. Cardiol. 51:300–306. 18206740 10.1016/j.jacc.2007.09.043

[phy213301-bib-0019] Priebe, H. J. , J. C. Heimann , and J. Hedley‐Whyte . 1980 Effects of renal and hepatic venous congestion on renal function in the presence of low and normal cardiac output in dogs. Circ. Res. 47:883–890. 7192184 10.1161/01.res.47.6.883

[phy213301-bib-0020] Richards, W. O. , W. Scovill , B. Shin , and W. Reed . 1983 Acute renal failure associated with increased intra‐abdominal pressure. Ann. Surg. 197:183–187. 6600601 10.1097/00000658-198302000-00010 PMC1353107

[phy213301-bib-0021] Schrier, R. W. 2007 Cardiorenal versus renocardiac syndrome: is there a difference? Nat. Clin. Pract. Nephrol. 3:637. 18033224 10.1038/ncpneph0673

[phy213301-bib-0022] Schrier, R. W. , D. Shchekochikhin , and P. Gines . 2012 Renal failure in cirrhosis: prerenal azotemia, hepatorenal syndrome and acute tubular necrosis. Nephrol. Dial. Transplant. 27:2625–2628. 22492830 10.1093/ndt/gfs067

[phy213301-bib-0023] Umgelter, A. , W. Reindl , M. Franzen , C. Lenhardt , W. Huber , and R. M. Schmid . 2009 Renal resistive index and renal function before and after paracentesis in patients with hepatorenal syndrome and tense ascites. Intensive Care Med. 35:152–156. 18802688 10.1007/s00134-008-1253-y

[phy213301-bib-0024] Winton, F. R. 1931 The influence of venous pressure on the isolated mammalian kidney. J. Physiol. 72:49–61. 16994199 10.1113/jphysiol.1931.sp002761 PMC1403105

[phy213301-bib-0025] Winton, F. R. 1951 Hydrostatic pressures affecting the flow of urine and blood in the kidney. Harvey Lect. Series 47:21–52. 13052263

[phy213301-bib-0026] Winton, F. R. 1964 Arterial, venous, intrarenal, and extrarenal pressure effects on renal blood flow. Circ. Res. 15(SUPPL):103–109. 14206289

